# Supplementary material for: Tuning Plant Promoters Using a Simple Split Luciferase Method to Assess Transcription Factor-DNA Interactions
Source: ACS Synth Biol. 2023 Oct 19;12(11):3482–6. doi: 10.1021/acssynbio.3c00094 (PMC10661027; doi:10.1021/acssynbio.3c00094)

Tuning plant promoters using a simple split luciferase method to assess transcription factor-DNA interactions.

Cai, Y-M[1], Witham, S[1], and Patron NJ[1]
1. Engineering Biology, Earlham Institute, Norwich Research Park, Norwich, NR4 7UZ, UK

Supporting Information

S1. Plasmids used in this study

Plasmids for transcription factor protein expression					
**Addgene#**	**Plasmid code**	**Description**	**Acceptor**	**Plasmid type**	**Source of plasmid**
196141	pEPYCeGM0001	AtTGA1:HiBiT	pDONR207	Gateway Entry	This study
196143	pEPYCeGM0009	AtNLP6:HiBiT	pDONR207	Gateway Entry	This study
196144	pEPYCeGM0010	AtNLP7:HiBiT	pDONR207	Gateway Entry	This study
196146	pEPYCeGM0012	AtNAC032:HiBiT	pDONR207	Gateway Entry	This study
196147	pEPYCeGM0022	AtARF18:HiBiT	pDONR207	Gateway Entry	This study
196148	pEPYCdKN0001	T7_9xHis:AtTGA1:HiBiT	pH9GW	Gateway Expression	This study
196150	pEPYCdKN0009	T7_9xHis:AtNLP6:HiBiT	pH9GW	Gateway Expression	This study
196151	pEPYCdKN0010	T7_9xHis:AtNLP7:HiBiT	pH9GW	Gateway Expression	This study
196153	pEPYCdKN0012	T7_9xHis:AtNAC032:HiBiT	pH9GW	Gateway Expression	This study
196154	pEPYCdKN0022	T7_9xHis:AtARF18:HiBiT	pH9GW	Gateway Expression	This study
	pH9GW	pDESTINATION LacI+T7_9xHIS	n/a	Gateway Destination	Paul O'Maille

Level 0 Phytobricks							
**Addgene#**	**Plasmid code**	**Part type**	**Description**	**Compatibility with Assembly Systems**	**Cloning overhang (top strand)**		**Source of plasmid**
					**5'**	**3'**	
196155	pEPYC0CM0515	PROM	MinSyn_301	MoClo, Loop, GB	GGAG	TACT	This study
196156	pEPYC0CM0519	PROM	MinSyn_302	MoClo, Loop, GB	GGAG	TACT	This study
196157	pEPYC0CM0520	PROM	MinSyn_303	MoClo, Loop, GB	GGAG	TACT	This study
196158	pEPYC0CM0522	PROM	MinSyn_305	MoClo, Loop, GB	GGAG	TACT	This study
196159	pEPYC0CM0513	PROM	MinSyn_308	MoClo, Loop, GB	GGAG	TACT	This study
196160	pEPYC0CM0528	PROM	MinSyn_310	MoClo, Loop, GB	GGAG	TACT	This study
196161	pEPYC0CM0529	PROM	MinSyn_311	MoClo, Loop, GB	GGAG	TACT	This study
154503	pEPYC0CM0035	PROM	MinSyn_000	MoClo, Loop, GB	GGAG	TACT	Cai et al., 2020
50255	pICH42211	PROM	AtuNOSp	MoClo, Loop, GB	GGAG	TACT	Engler et al., 2014
50272	pICH85281	PROM	AtuMASp	MoClo, Loop, GB	GGAG	TACT	Engler et al., 2014
50285	pICH41402	5UTR	TMVΩ	MoClo, Loop, GB	TACT	AATG	Engler et al., 2014
154594	pEPAS0CM0008	CDS	LucF	MoClo, Loop, GB	AATG	TTCG	Cai et al., 2020
154595	pEPYC0CM0133	CDS	LucN	MoClo, Loop, GB	AATG	TTCG	Cai et al., 2020
50308	pICSL50007	CTAG	FLAG tag	MoClo, Loop, GB	TTCG	GCTT	Engler et al., 2014
50343	pICH41432	3UTR+TERM	AtuOCSt	MoClo, Loop, GB	GCTT	CGCT	Engler et al., 2014
50339	pICH41421	3UTR+TERM	AtuNOSt	MoClo, Loop, GB	GCTT	CGCT	Engler et al., 2014

Level 1 Assemblies					
**Addgene#**	**Plasmid code**	**Plasmid type**	**Contents**	**Acceptor**	**Source of plasmid**
196166	pEPYC1CB0597	Plant expression (promoter:LucF)	MinSyn_301 (pEPYC0CM0515)_TMV(pICH41402)_LucF (pEPAS0CM0008)_FLAG(pICSL50007)_ocsT(pICH41432)	pICH47732 (Addgene 48000)	This study
196167	pEPYC1CB0601	Plant expression (promoter:LucF)	MinSyn_302 (pEPYC0CM0519)_TMV(pICH41402)_LucF (pEPAS0CM0008)_FLAG(pICSL50007)_ocsT(pICH41432)	pICH47732 (Addgene 48000)	This study
196168	pEPYC1CB0602	Plant expression (promoter:LucF)	MinSyn_303 (pEPYC0CM0520)_TMV(pICH41402)_LucF (pEPAS0CM0008)_FLAG(pICSL50007)_ocsT(pICH41432)	pICH47732 (Addgene 48000)	This study
196169	pEPYC1CB0604	Plant expression (promoter:LucF)	MinSyn_305 (pEPYC0CM0522)_TMV(pICH41402)_LucF (pEPAS0CM0008)_FLAG(pICSL50007)_ocsT(pICH41432)	pICH47732 (Addgene 48000)	This study
196170	pEPYC1CB0595	Plant expression (promoter:LucF)	MinSyn_308 (pEPYC0CM0513)_TMV(pICH41402)_LucF (pEPAS0CM0008)_FLAG(pICSL50007)_ocsT(pICH41432)	pICH47732 (Addgene 48000)	This study
196171	pEPYC1CB0607	Plant expression (promoter:LucF)	MinSyn_310 (pEPYC0CM0528)_TMV(pICH41402)_LucF (pEPAS0CM0008)_FLAG(pICSL50007)_ocsT(pICH41432)	pICH47732 (Addgene 48000)	This study
196172	pEPYC1CB0608	Plant expression (promoter:LucF)	MinSyn_311 (pEPYC0CM0529)_TMV(pICH41402)_LucF (pEPAS0CM0008)_FLAG(pICSL50007)_ocsT(pICH41432)	pICH47732 (Addgene 48000)	This study
154630	pEPYC1CB0007	Plant expression (promoter:LucF)	MinSyn_000 (pEPYC0CM0035)_TMV(pICH41402)_LucF (pEPAS0CM0008)_FLAG(pICSL50007)_ocsT(pICH41432)	pICH47732 (Addgene 48000)	Cai et al., 2020
154654	pEPYC1CB0197	Plant expression calibrator (promoter:LucN)	NOSp(pICH42211)_TMV(pICH41402)_LucN (pEPAS0CM0133)_FLAG(pICSL50007)_nosT(pICH41421)	pICH47732 (Addgene 48000)	Cai et al., 2020
154655	pEPYC1CB0199	Plant expression experiment calibrator (promoter:LucF)	MASp(pICH85281)_TMV(pICH41402)_LucF (pEPAS0CM0008)_FLAG(pICSL50007)_ocsT(pICH41432) (experiment normaliser)	pICH47732 (Addgene 48000)	Cai et al., 2020

S2. Sequences of DNA probes

Name and source	TF	Oligo ID	Sequence
Random (rnd)	NONE	ptoz179_PS_NC_F	TAGCGAAGTACGATCCCATGAAGACGCTGGGTTTACATGGAATGGTGCTTCTGTTCTAACAGGCTAGGATATAAGGCCATCACGCAGTA
		ptoz180_PS_NC_R	TACTGCGTGATGGCCTTATATCCTAGCCTGTTAGAACAGAAGCACCATTCCCATGTAAACCCAGCGTCTTCATGGGATCGTACTTCGCTA
CaMV35s	TGA1	YMC175	ATGAAGACGCTGGGTTTACATGGGAATGGCTGACGTAAGGGATGACGCACATGCTTCTGTTCTAACAGGCTAGGATATAA
		YMC176	TTATATCCTAGCCTGTTAGAACAGAAGCATGTGCGTCATCCCTTACGTCAGCCATTCCCATGTAAACCCAGCGTCTTCAT
NIR1	NLP6/ NLP7	ptoz177_PS_PC_F	TAGCGAAGTACGATCCCATCAAAGAGAAACAACTTGACCCTTTACATTGCTCAAGAGCTCATCTCTTCCCTCTACGGCCATCACGCAGTA
		ptoz178_PS_PC_R	TACTGCGTGATGGCCGTAGAGGGAAGAGATGAGCTCTTGAGCAATGTAAAGGGTCAAGTTGTTTCTCTTTGATGGGATCGTACTTCGCTA
ANAC032SO1	ANAC032	ptoz369_PC2_ANAC032_F	TAGCGAAGTACGATCCCATGAAGACGGAGGTAAGCAAATTGATCACGCAACTGGTGGATATAAGGCCATCACGCAGTA
		ptoz370_PC2_ANAC032_R	TACTGCGTGATGGCCTTATATCCACCAGTTGCGTGATCAATTTGCTTACCTCCGTCTTCATGGGATCGTACTTCGCTA
DR5(7X)	ARF18	DR5F	CCTTTTGTCTCCCTTTTGTCTCCCTTTTGTCTCCCTTTTGTCTCCCTTTTGTCTCCCTTTTGTCTCCCTTTTGTCTC
		DR5R	GAGACAAAAGGGAGACAAAAGGGAGACAAAAGGGAGACAAAAGGGAGACAAAAGGGAGACAAAAGGGAGACAAAAGG
TFBS_07 AT3G14060	TGA1	YMC183	TCCCATGAAGACGCTGGGTTTACATGGGAATGCATGACATCAACGTGGTGCTTCTGTTCTAACAGGCTAGGATATAAGGC
		YMC184	GCCTTATATCCTAGCCTGTTAGAACAGAAGCACCACGTTGATGTCATGCATTCCCATGTAAACCCAGCGTCTTCATGGGA
TFBS_05 AT3G14205	TGA1	YMC187	TCCCATGAAGACGCTGGGTTTACATGGGAATGCATTACATCATCATAGTGCTTCTGTTCTAACAGGCTAGGATATAAGGC
		YMC188	GCCTTATATCCTAGCCTGTTAGAACAGAAGCACTATGATGATGTAATGCATTCCCATGTAAACCCAGCGTCTTCATGGGA
TFBS_09 AT1G68490	TGA1	YMC189	TCCCATGAAGACGCTGGGTTTACATGGGAATGTGTCACATCAGCATAGTGCTTCTGTTCTAACAGGCTAGGATATAAGGC
		YMC190	GCCTTATATCCTAGCCTGTTAGAACAGAAGCACTATGCTGATGTGACACATTCCCATGTAAACCCAGCGTCTTCATGGGA
TFBS_02 AT1G77450	TGA1	ptoz329_PS_ANAC032_3F	TAGCGAAGTACGATCCCGGACCGCTACATTCCAAATAGTCTGACGTAAGCAATGACAAAACTCACCTACATGGCCATCACGCAGTA
		ptoz330_PS_ANAC032_3R	TACTGCGTGATGGCCATGTAGGTGAGTTTTGTCATTGCTTACGTCAGACTATTTGGAATGTAGCGGTCCGGGATCGTACTTCGCTA
TFBS_12 AT1G64530	TGA1	ptoz307_PS_NLP6_2F	TAGCGAAGTACGATCCCAACACGCACTTAGTGCCTAATTACGTCTTAATTTAATATTTTTTAAAGCGGCCATCACGCAGTA
		ptoz308_PS_NLP6_2R	TACTGCGTGATGGCCGCTTTAAAAAATATTAAATTAAGACGTAATTAGGCACTAAGTGCGTGTTGGGATCGTACTTCGCTA
TFBS_14 AT3G61830	TGA1	ptoz555_Pr_104_PS_ARF18_3F	TAGCGAAGTACGATCCCTGAGTTTCCTTTTTAGCGATGACATGATAAAACAAAAAACAACAATTTGGCCATCACGCAGTA
		ptoz556_Pr_104_PS_ARF18_3R	TACTGCGTGATGGCCAAATTGTTGTTTTTTGTTTTATCATGTCATCGCTAAAAAGGAAACTCAGGGATCGTACTTCGCTA

S3. Example data and analysis

	0 nM	1.5 fM	5 nM	15 nM	2x free probe 15 nM	5x free probe 15 nM protein	10x free probe 15 nM protein	rnd probe 15 nM protein
HiBit Rep1	579	224535	567146	804307	49390	28536	23612	324075
HiBit Rep2	600	248884	560068	940370	63746	42783	26734	397371
HiBit Rep3	421	306935	793533	1192230	119946	39637	35014	398011
Pico Rep 1	16859	16859	16859	16859	16859	16859	16859	20810
Pico Rep 2	9102	9102	9102	9102	9102	9102	9102	15166
Pico Rep 3	12382	12382	12382	12382	12382	12382	12382	9305
mean	12781	12781	12781	12781	12781	12781	12781	15093.66667
HiBit/Pico Rep1	0.04530162	17.5678742	44.3741491	62.9298959	3.864329865	2.232689148	1.847429779	21.470926
HiBit/Pico Rep2	0.04694468	19.4729677	43.8203583	73.5756201	4.987559659	3.347390658	2.091698615	26.3270025
HiBit/Pico Rep3	0.03293952	24.0149441	62.0869259	93.2814334	9.384711681	3.101244034	2.739535248	26.36940439
mean	0.04172861	20.3519286	50.0938111	76.5956498	6.078867068	2.893774613	2.226221214	24.72244429
2xSE	0.00884013	3.8245883	11.997376	17.7818031	3.36885099	0.676187878	0.532334843	3.251610454
Tukey HSD	d	c	b	a	cd	cd	cd	c

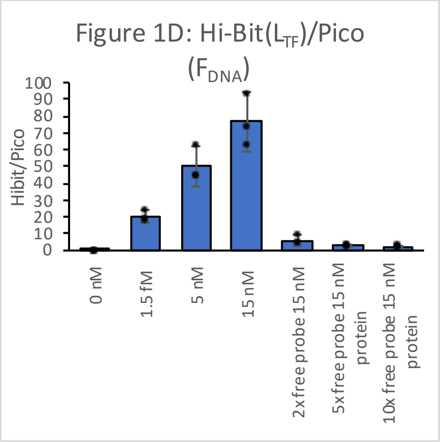

	TGA probe	Rnd Probe
Rep1 normalised to rnd (rQ)	2.54545607	0.868479093
Rep2 Normalised to rnd (rQ)	2.97606576	1.064902895
Rep3 Normalised to rnd (rQ)	3.77314768	1.066618012
mean	3.09822317	1
2xSE	0.71925748	0.131524635

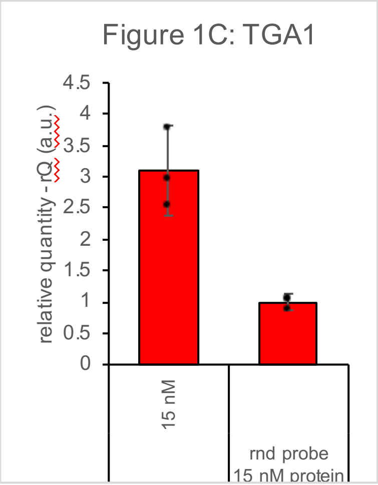

Supplement: Supplementary file 1 — sb3c00094_si_001.pdf [file sb3c00094_si_001.pdf]
